# Remodeling of Kv7.1 and Kv7.5 Expression in Vascular Tumors

**DOI:** 10.3390/ijms21176019

**Published:** 2020-08-21

**Authors:** Clara Serrano-Novillo, Anna Oliveras, Joan Carles Ferreres, Enric Condom, Antonio Felipe

**Affiliations:** 1 Molecular Physiology Laboratory, Department de Bioquímica i Biomedicina Molecular, Institut de Biomedicina (IBUB), Universitat de Barcelona, 08028 Barcelona, Spain; clara.serrano.n@gmail.com (C.S.-N.); aolivema@gmail.com (A.O.); 2 Consorci Corporació Sanitària Parc Taulí-Parc Taulí Hospital Universitari, Universitat Autónoma de Barcelona, 08208 Sabadell, Spain; joancfp@gmail.com; 3 Institut d’Investigació Biomèdica de Bellvitge (IDIBELL), Hospital Universitari de Bellvitge, 08907 L’Hospitalet de Llobregat, Spain; ecm@bellvitgehospital.cat; 4 Departament de Bioquímica i Biomedicina Molecular, Universitat de Barcelona, Avda. Diagonal 643, 08028 Barcelona, Spain

**Keywords:** potassium channels, blood vessels, neoplasia, cancer

## Abstract

Voltage-dependent potassium (Kv) channels contribute to the excitability of nerves and muscles. In addition, Kv participates in several cell functions, including cell cycle progression and proliferation. Kv channel remodeling has been associated with neoplastic cell growth and cancer. Kv7 channels are expressed in blood vessels, and they participate in the maintenance of vascular tone and are implicated in myocyte proliferation. Although evidence links Kv7 remodeling to different types of cancer, its expression in vascular tumors has never been studied. Endothelium-derived vascular neoplasms range from indolent lesions to highly aggressive and metastasizing cancers. Here, we show that Kv7.1 and Kv7.5 are evenly distributed in tunicas as well as the endothelium of healthy veins and arteries. The layered structure of vessels is lost in vascular tumors. By studying eight vascular tumors with different origins and characteristics, we found that Kv7.1 and Kv7.5 expression was changed in vascular cancers. While both channels were generally downregulated, Kv7.5 expression was clearly correlated with neoplastic malignancy. The vascular tumors did not contract; therefore, the role of Kv7 channels is probably related to proliferation rather than controlling vascular tone. Our results identify vascular Kv7 channels as targets for cancer detection and anticancer therapies.

## 1. Introduction

Voltage-dependent potassium channels (Kv) control the action potential of nerves and muscles. In addition, Kv channels contribute to the membrane potential by participating in the electrochemical gradient. Furthermore, changes in the membrane potential are crucial during cell cycle progression and proliferation. Therefore, Kv channels are actively involved in physiological and neoplastic cell proliferation [1,2,3,4].

Vascular tumors result from the pathological proliferation of vascular endothelial cells with variable growth capacity. Healthy blood vessels organize in stratified cell layers named tunica. While tunica media mainly contains smooth muscle, the endothelium forms the tunica intima. Endothelial tumors, which are heterogenous in both histology and behavior, are classified as malformations or neoplasms depending on the presumed origin of the malignancy. Many tumors undergo spontaneous involution, others proliferate and become static, and a small subset are locally aggressive and metastasize [5,6].

Potassium channels from the Kv7 family participate in the maintenance of vascular tone. While Kv7 channel activators trigger vasodilation, inhibitors contract vessels [7]. The Kv7 family consists of five members (Kv7.1–Kv7.5), which generate outward delayed rectifying potassium currents and have a central role in regulating cell excitability. Kv7.1, Kv7.4 and Kv7.5 are expressed in vascular smooth muscle. Kv7.4 and Kv7.5 participate in the contractility of vascular cells [7]. Additionally, PKC (protein kinase C) suppresses Kv7.5 currents upon vessel constriction in rat aortas or mesenteric arteries. This depolarizes the membrane, opening Ca2+ channels and promoting muscle contraction [8]. In addition, Kv7.4, alone or in heterotetramers with Kv7.5, contributes to vasodilation [9]. Moreover, a variety of veins and arteries express Kv7.1, suggesting a possible role in smooth muscle contraction [10]. However, the contribution of Kv7.1 is an open debate. Outward K+ currents are linked to Kv7.1 in murine veins [11] and its expression has been confirmed in human arteries; however, Kv7.1 antagonist treatment did not alter vascular tone [12]. Evidence attributes the absence of Kv7.1 functions to the presence of auxiliary KCNE subunits, such as KCNE4 and KCNE5, which inhibit Kv7.1 currents and are often coexpressed with the channel in blood vessels [13]. Another possibility is the heterotetramerization of the channel. Kv7.1 associates with Kv7.5 in vascular myocytes, which alters the biophysical properties of the channel [14].

As mentioned above, Kv channels participate in regulating membrane potential, and they facilitate cell cycle progression to promote cell growth [1,2,3]. Thus, Kv7.1 and Kv7.5 are implicated in myocyte proliferation. Kv7.1 is remodeled in some tumors and Kv7.5 blockers halt proliferation. Kv7.1 increases in colon cancer and lung adenocarcinoma as well as in seminoma and other germ cell tumors [15,16,17]. On the other hand, even though Kv7.5 contributes to myoblast proliferation [18] and vascular physiology, it has not been studied in tumors other than osteosarcoma [19].

In the present work, we studied different vascular and perivascular tumors categorized as benign, intermediate and malignant. Tumors were histologically classified according to the 5th edition of the World Health Organization (WHO) classification of soft tissue tumors [20]. The samples are from the following types: angioma, glomus tumor, Kaposi’s sarcoma, epithelioid haemangioendothelioma and angiosarcoma. Following WHO classification, angiomas were categorized as either benign endothelial neoplasms or malformative lesions, which are often present at birth. Kaposi’s sarcoma is a locally aggressive neoplasia. Epithelioid haemangioendothelioma and angiosarcoma are fully malignant tumors with a high proliferative index. Our data show that while Kv7.1 expression is mostly downregulated with no apparent correlation with tumor aggressiveness, the expression of Kv7.5 is correlated with malignancy.

## 2. Results

### 2.1. Expression of Kv7.1 and Kv7.5 in Human Blood Vessels

**Figure 1:**
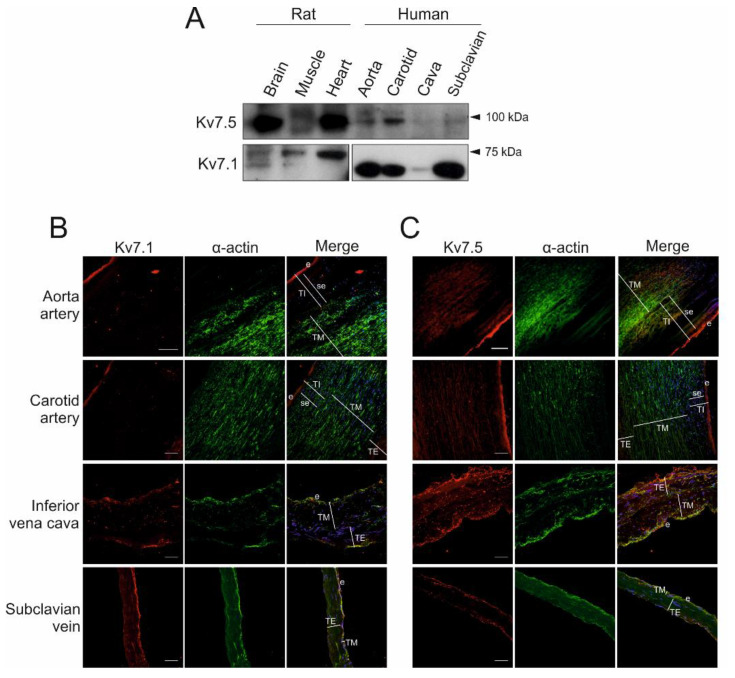
Voltage-dependent potassium (Kv)7.1 and Kv7.5 are differentially expressed in human blood vessels. (**A**) Membrane protein extracts were obtained from several human blood vessels (aorta and carotid arteries and inferior cava and subclavian veins) and rat tissue samples (brain, skeletal muscle and heart). (**B** and **C**) Fresh human samples were obtained and processed as described in the Materials and Methods. Sections were stained with anti-Kv7.1 (**B**) or anti-Kv7.5 (**C**) antibodies. Then, α-actin was used to identify muscle structures. Red, Kv7.1 (**B**) and Kv7.5 (**C**) channels; green, α-actin; merge, superimposed channel, α-actin and nuclear DAPI staining (in blue). TI: tunica intima; TM: tunica media; TE: tunica externa; e: endothelial layer; se: subendothelial layer. Scale bars: 50 μm in cava and subclavian veins; 100 μm in carotid artery; 250 μm in aorta artery.

Members of the Kv7 family are mainly expressed in nerves and muscles. While Kv7.1 has a critical role in heart physiology, Kv7.5 contributes to the neuronal M-current [21]. The channels are similarly regulated during muscle proliferation, and they both exhibit human cardiovascular smooth muscle vessel expression (Figure 1). Thus, Kv7.1 and Kv7.5 are differentially expressed in arteries (aorta and carotid) and veins (inferior vena cava and subclavian). Based on these results, Kv7.5 seems more abundant in arteries than veins. Rat brain, heart and skeletal muscle were used as positive controls. Slight differences in molecular weight were observed due to isoform variabilities between tissues and species (Figure 1A).

Histological analysis and fluorescent staining of human veins and arteries further supported Kv7.1 and Kv7.5 expression in blood vessels (Figure 1B,C). However, the staining was not evenly distributed, as demonstrated by α-actin results. Although both channels are expressed in the tunica media vascular smooth muscle in both veins and arteries, the signal was more intense in the endothelium within the tunica intima. Negative control experiments, performed in all cases, demonstrated that channel staining was specific (Figure S1 (Supplementary Materials)).

### 2.2. Kv Channel Expression in Vascular Tumors

Twenty-seven cases of eight different vascular tumors were initially divided into two groups depending on the specimen preparation. Then, paraffin-embedded and optimal cutting temperature compound (OCT) samples were analyzed. The paraffin-embedded group contained cavernous angioma, capillary angioma, Kaposi’s sarcoma, angiosarcoma and glomus tumors. The first four are derived from endothelial cells and exhibit different grades of malignancy, from benign or indolent to highly malignant. Glomus tumors are derived from modified smooth muscle cells of the normal glomus body (glomus cells) surrounding the vessel walls. The OCT-embedded group included three malignant, highly metastatic and very rare tumors: cutaneous angiosarcoma, epithelioid hemangioendothelioma and testicular teratoma-derived angiosarcoma. Because neoplasms of these types are rare, the sample availability was very limited but considered necessary to provide essential information. Considering the distinct process required because of their different embedding and the limitation of samples for the analysis of the OCT-embedded tumors, the two groups were analyzed separately.

**Figure 2:**
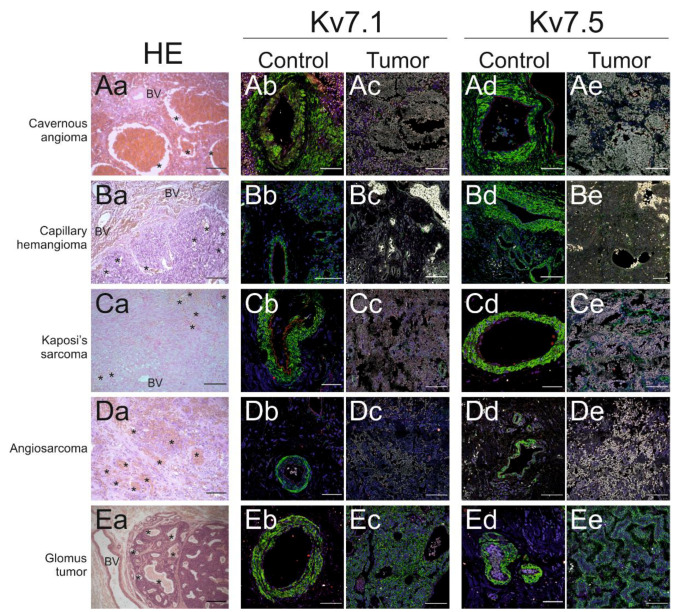
Kv7.1 and Kv7.5 expression in paraffin-embedded vascular tumors. Consecutive sections of vascular tumor samples were stained with hematoxylin-eosin (HE) and were immunofluorescently labeled for Kv7.1 and Kv7.5. (**Aa**–**Ae**) Cavernous angioma. (**Ba**–**Be**) Capillary hemangioma. (**Ca**–**Ce**) Kaposi’s sarcoma. (**Da**–**De**) Angiosarcoma. (**Ea**–**Ee**) Glomus tumor. HE was used to identify structures. BV: healthy blood vessels; *: tumoral blood vessels. Scale bars: 50 μm, capillary angioma; 100 μm, cavernous angioma, Kaposi’s sarcoma and angiosarcoma; 200 μm, glomus tumor. Representative immunolabeling images showing merged Kv7.1 in healthy (**Ab**,**Bb**,**Cb**,**Db**,**Eb**) and tumor (**Ac**,**Bc**,**Cc**,**Dc**,**Ec**) biopsies. Representative immunolabeling images showing merged Kv7.5 in healthy (Ad, Bd, Cd, Dd, Ed) and tumor (**Ae**,**Be**,**Ce**,**De**,**Ee**) biopsies. Red, channels; green, α-actin (muscle); blue, DAPI nuclear staining; gray, far red detection of red blood cells showing high autofluorescence Scale bars: 100 μm in cavernous and capillary angioma; 80 μm in Kaposi’s sarcoma, angiosarcoma and glomus tumor.

Paraffin-embedded samples were stained with hematoxylin-eosin to enable a detailed morphological evaluation (Figure 2Aa,Ba,Ca,Da,Ea,Fa).

Cavernous haemangioma (Cv) is a benign vascular lesion that is often a malformation in origin (Figure 2Aa). This representative specimen presented a sponge-like appearance due to dilated and congestive vessels lined by flattened endothelial cells with no visible muscle layer.

Lobular capillary haemangioma (Cp) is a reactive vascular lesion that mostly appears in the skin or mucosal surfaces (Figure 2Ba). This indolent nonneoplastic lesion was made of small capillary-like vessels arising from larger vessels, and it exhibited a lobular appearance.

Kaposi’s sarcoma (KS), mostly located in the dermis (Figure 2Ca), grew as fascicles of spindle cells; there was a prominent surrounding haemorrhage and small slit-like spaces containing erythrocytes.

Angiosarcoma (As) is a malignant neoplasia arising from the endothelial cells lining blood or lymphatic vessels (Figure 2Da). This highly aggressive and proliferative sample was subcutaneous and exhibited many mitotic cells. Spindle and epithelioid atypical cells formed solid masses or bundles, and there were irregular and anastomosing cavities containing red blood cells. There was marked cytological atypia and pleomorphism with a high mitotic index.

Glomus tumor (GT) is a rare indolent neoplasm located in the dermis (Figure 2Ea). This tumor does not originate from endothelial cells but from glomus cells, which are a type of a modified smooth muscle cell with thermoregulatory function. Tumors grew as nodules and contained blood vessels.

Kv7.1 and Kv7.5 staining was studied in the tumors (Figure 2Ab–Ee). Confocal microscopy imaging at low magnification was used to obtain an image of the whole sample. Next, several nontumor and tumor areas within the same sample were identified using a screening system. As mentioned above, the blood vessel endothelium expressed Kv7.1 and Kv7.5 in healthy vessels with a normal layer structure. Channel staining within the tumor areas was consistently disorganized. Neoplastic vessels showed no layers. To evaluate changes caused by neoplastic transformation, healthy expression was compared to affected areas. Kv7.1 and Kv7.5 expression levels were calculated from their fluorescence intensity in healthy and tumor areas from each specimen. Channel expression levels from different individuals revealed high variability among patients. Therefore, the data were normalized, and the relative expression was compared (Figure S2 (Supplementary Materials)). Because intrinsic patient variability in healthy areas arose from different types of analyzed vessels, including veins and arteries, both were considered. However, the staining was uniform in tumor areas. Our data revealed that Kv7.5, and Kv7.1 to a greater extent, exhibited decreased expression in vascular tumors.

**Figure 3:**
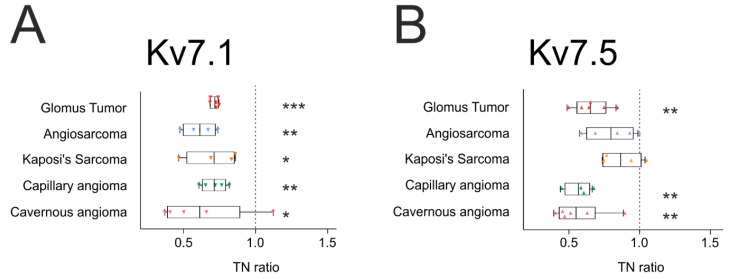
Tumor-to-normal (TN) Kv7.1 and Kv7.5 ratios. TN ratios were calculated as described in the Materials and Methods from protein expression levels in immunofluorescence assays, and the data are represented in a box-and-whisker plot. The analysis was performed for Kv7.1 (**A**) and Kv7.5 (**B**). The reference value (1) is indicated by a dotted vertical line. Boxes represent the interquartile range (IQR: 25–75%) of the values. The inside-box line represents the mean of each group. Minimum and maximum values are indicated outside the box. Cavernous angioma, in magenta; Capillary angioma, in green; Kaposi’s sarcoma, in orange; Angiosarcoma, in blue; Glomus tumor, in red. * *p* < 0.05, ** *p* < 0.01 and *** *p* < 0.001 vs. reference value (one sample *t*-test statistics).

The tumor-to-normal ratio (TN ratio) was calculated (Figure 3). This index represented the relation between the expression in tumors and the expression in healthy areas. Ratios above one indicated an increase in expression, whereas ratios below one corresponded to a downregulation of the channel in cancerous areas. Data confirmed that Kv7.1 and Kv7.5 were remodeled in human vascular tumors. Kv7.1 showed significant changes (Figure 3A). TN ratios were similar, with a 30–40% reduction in all tumors. While glomus tumors appeared to be very homogeneous, cavernous angiomas were highly heterogeneous. Unlike Kv7.1, Kv7.5 exhibited variability among tumors (Figure 3B). Although the overall results showed downregulation of Kv7.5, only the changes in capillary and cavernous angioma and glomus tumors were significant. Interestingly, malignant KS and angiosarcoma exhibited only minor Kv7.5 changes. In contrast, angiomas, which are sometimes considered vascular malformations rather than neoplasms, exhibited consistently lower Kv7.5 expression.

Neoplastic vessels were also analyzed. However, changes in Kv expression were not related to any anatomical and histological features of tumor vessels. Thus, no correlation between the size/shape of the neoangiogenic vessels and Kv7.1 or Kv7.5 alterations was found (data not shown). Together, our data demonstrated that Kv7.1 and Kv7.5 remodeled expression during vascular neoplastic changes, mainly through exhibiting reduced expression.

**Figure 4:**
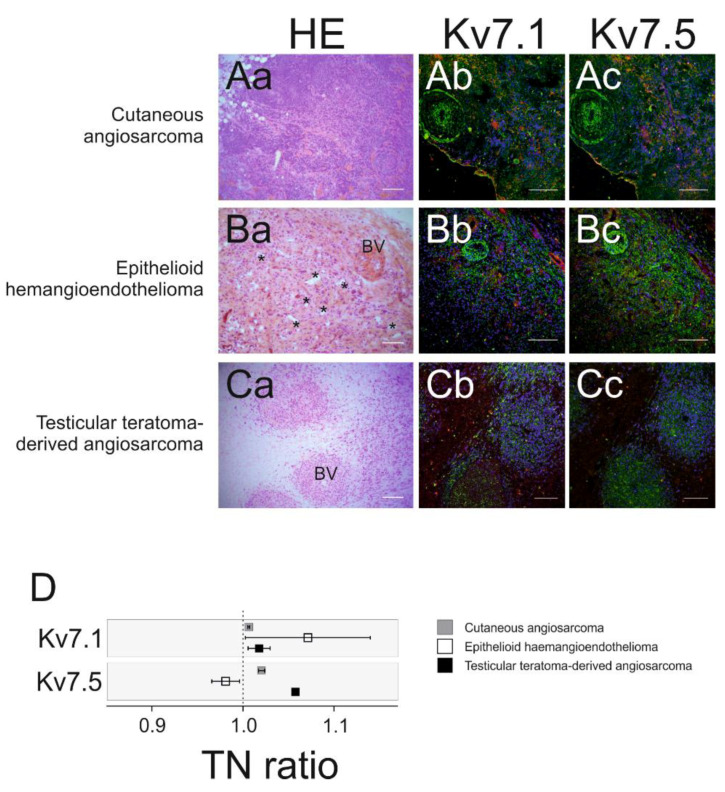
Kv7.1 and Kv7.5 expression in optimal cutting temperature compound (OCT)-embedded malignant vascular tumors. Consecutive sections of vascular tumor samples were stained by hematoxylin-eosin (HE) and immunofluorescent labeling for Kv7.1 and Kv7.5. (**Aa**–**Ac**) Cutaneous angiosarcoma. (**Ba**–**Bc**) Epithelioid haemangioendothelioma. (**Ca**–**Cc**) Testicular teratoma-derived angiosarcoma. (**Aa**,**Ba**,**Ca**) HE was used to identify structures. BV: healthy blood vessels; *: tumoral blood vessels. Scale bars 150 μm. Representative immunolabeling images showing merged Kv7.1 (**Ab**,**Bb**,**Cb**) and Kv7.5 (**Ac**,**Bc**,**Cc**) and tumor biopsies. Red, channels; green, α-actin (muscle); blue, DAPI nuclear staining. Scale bars 150 μm. (**D**) Tumor-to-normal (TN) ratios, in arbitrary units, were calculated from the protein expression levels obtained in immunofluorescence assays and represented in a forest plot. The analysis was performed for Kv7.1 (**top**) and Kv7.5 (**bottom**) channels, plotted in horizontal bars. The reference value (1) is indicated by a dotted line. Squares represent the mean ± SEM of the TN ratio for each patient and tumor. Gray, cutaneous angiosarcoma. White, epithelioid haemangioendothelioma. Black, testicular teratoma-derived angiosarcoma. Mean ± SEM, only represented when multiple samples and regions of interest (ROIs) were available.

The second group of tumors studied included OCT frozen samples from the following malignant rare blood vessel tumors: cutaneous angiosarcoma, epithelioid haemangioendothelioma and angiosarcoma derived from a highly proliferative and aggressive testicular teratoma. These cancers are characterized as the most malignant group of vascular tumors. The OCT samples were also stained with hematoxylin-eosin to enable identification of their main histological features (Figure 4Aa,Ba,Ca).

Cutaneous angiosarcoma (CAs). (Figure 4Aa). Tumoral cells presented elongated nuclei and created bundles, enabling solid and infiltrative growth, that dissected the dermal collagen and subcutaneous fat.

This epithelioid haemangioendothelioma (EHE) was located in the liver (Figure 4Ba). The tumor was composed of epithelioid large cells and the remaining collagenous stroma. This structure endowed the tumor with a mixed matrix composition with dense areas, containing high nuclear concentration, and paucicellular areas, containing mainly stroma. The lumen of small vessel appeared as small vacuoles in between one or two tumoral cells, and they were characterized by reduced cytoplasm and atypical nuclei.

Testicular teratoma-derived angiosarcoma (TAs). This representative sample was a primary testicular angiosarcoma and mature teratoma (Figure 4Ca). Histologically similar to cutaneous angiosarcoma. Thus, this biopsy depicted a solid architectural pattern with no neoangiogenic lumens and fusiform tumoral bundles. This highly proliferative and malignant tumor contained necrotic areas.

Analysis of Kv7.1 and Kv7.5 expression was performed in areas containing healthy and tumoral blood vessels (Figure 4Ab–Cc). Confocal images were captured, Kv7.1 and Kv7.5 fluorescence intensities were analyzed, and TN ratios were calculated to compare tumoral and control areas (Figure 4D). Multiple regions of interest (ROIs) were evaluated from each tumor. However, as mentioned above, tumor availability was very limited due to their extremely low prevalence. Although limited information can be obtained at this point, the TN ratios revealed minor Kv7 expression changes in most of the sections.

### 2.3. Kv7.1 and Kv7.5 Expression Correlates with Tumor Malignancy

**Figure 5:**
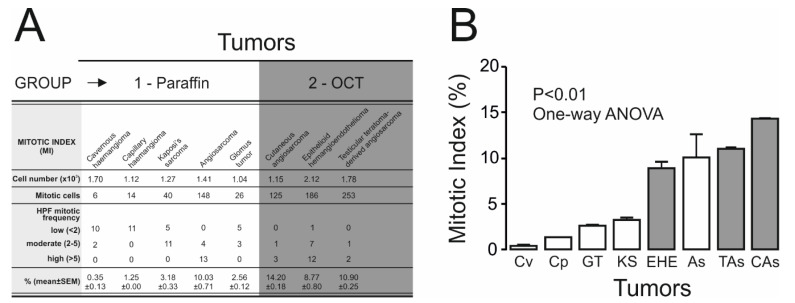
Mitotic index of tumors. Histological patterns were identified, and the mitotic index (MI) was evaluated from the hematoxylin-eosin stained samples. (**A**) The MI was calculated by counting all mitotic events present in 10 high-power fields (HPF) at 400× magnification. HPF, based on the frequency of mitosis, were classified as low (<2 mitosis in 10 HPF), moderate (2–5 mitosis in 10 HPF) or high (>5 mitosis in 10 HPF). The % values indicate the percentage of mitotic cells compared with the total number of cells evaluated. OCT-frozen malignant tumor samples are identified by a dark gray box. (**B**) The MI plotted against the tumor type. Values from panel A were plotted versus tumors, and the malignancy for each cancer was determined. While white bars represent paraffin-embedded samples, gray bars indicate OCT samples. Values are represented as the mean ± SEM; *p* < 0.01 (one-way ANOVA).

Kv7.1 and Kv7.5 have been linked to muscle proliferation [18], and our data demonstrated that both channels are remodeled in vascular cancers. Therefore, we next wanted to decipher whether this phenotype followed a relationship with malignancy. Tumor malignancy and mitotic index (MI) were evaluated by histological properties (Figure 5A). Because higher cell division rates are commonly associated with more malignant phenotypes, the highest MI values typically correspond with malignant rare tumors. Therefore, although the information yielded by these rare cases should be taken with caution because few samples were available, they provide qualitatively important information that accurately represent these samples (Figure 5B).

**Figure 6:**
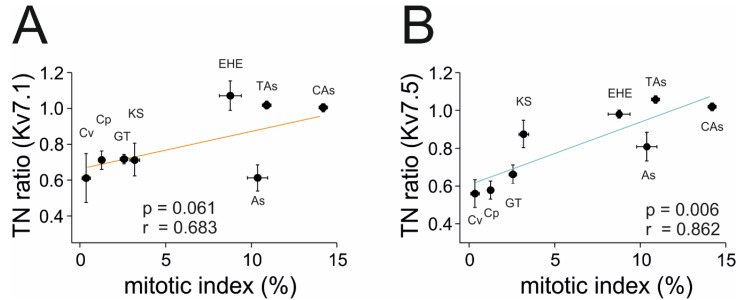
Kv7.1 and Kv7.5 expression according to tumor malignancy. Kv7.1 (**A**) and Kv7.5 (**B**) TN ratios (tumor-to-normal ratios) plotted against their mitotic index as a marker of the clinical aggressiveness of vascular tumors. A Pearson’s correlation coefficient of r = 0.683 with *p* = 0.061 indicates no apparent correlation between Kv7.1 expression and tumor aggressiveness. A Pearson’s correlation coefficient of r = 0.862 with *p* = 0.006 indicates a direct correlation between Kv7.5 expression and tumor aggressiveness. Values are the mean ± SEM. Cv: cavernous angioma; Cp: capillary angioma; GT: glomus tumor; KS: Kaposi’s sarcoma; EHE: epithelioid haemangioendothelioma; As: angiosarcoma; TAs: testicular teratoma-derived angiosarcoma; and CAs: cutaneous angiosarcoma.

MI was used to rank the tumor malignancy, and we wondered whether it had any relationship to Kv7.1 and Kv7.5 expression. TN ratios were plotted against the malignancy of tumors, and Pearson’s correlation coefficient was calculated (Figure 6). As mentioned above, indolent tumors tended to exhibit downregulated Kv7.1 and Kv7.5 expression, whereas highly proliferative tumors maintained channel levels similar to those of healthy biopsies. Thus, Kv7.5 expression, and to a lesser extent Kv7.1 expression, was tightly correlated with the clinical aggressiveness of the tumor. Pearson’s correlation coefficients were r = 0.683 (*p* = 0.061) and r = 0.862 (*p* = 0.006) for Kv7.1 and Kv7.5, respectively. Only Kv7.5 reached statistically significant values (Figure 6B), but this was mainly due to the distortion generated by a unique patient with a cavernous angioma that uniquely exhibited increased rather than decreased Kv7.1 expression (Figure S2A (Supplementary Materials)). However, a clear difference was observed between malignant tumors, EHE, TAs and CAs, and the rest of the samples. Therefore, higher levels of Kv7.1 and Kv7.5 expression corresponded with the malignancy of vascular tumors.

## 3. Discussion

Kv channels are crucial for the cardiovascular system. Kv7.1 and Kv7.5 participate in vascular smooth muscle tone in the control of blood pressure [22]. In addition, Kv channels, which promote cell cycle progression, are involved in physiological and neoplastic cell growth. Many channels are under tight control during proliferation, as they can control carcinogenesis or can be remodeled because of cancer [1,2,3]. Thus, the final fate of these channels may result in aberrant function, which identifies these proteins either as the origin or as markers of carcinogenesis. During cell cycle regulation, Kv7.1 and Kv7.5 are essential for the cardiovascular system. Therefore, understanding channel physiology and expression are vital for understanding neoplastic remodeling and adaptation in vascular tumors. Here, we revealed that Kv7.1 and Kv7.5 are remodeled during blood vessel carcinogenesis. The well-structured layered composition of veins and arteries switched to a heterogeneous and amorphous endothelial-derived tumoral mass containing vessels with no layer structure and undefined transportation functions. Under these circumstances, Kv7.1 and Kv7.5 tend to exhibit decreased expression in tumors. Furthermore, the malignancy of the tumor correlated with Kv expression. This could have two alternative implications when considering these proteins for cancer detection and prevention: (i) Kv7 channels are no longer needed to control the vascular tone in indolent tumors because healthy blood vessels disappear or, as previously demonstrated, (ii) Kv7.1 and Kv7.5 play a role in the cell cycle progression of muscle cells [18]. Therefore, highly proliferative cancer cells maintain active channels that help tumor cells survive and divide.

The physiological relevance of Kv7 channels is such that alterations in channel expression trigger major diseases [23]. Thus, dysregulation of cardiac Kv7.1, often related to a gain-of-function phenotype, triggers long QT syndrome 1 [24]. In addition, defects in surface targeting of Kv7.2 or Kv7.3 are responsible for neuronal disorders such as epilepsy [25]. Finally, Kv7.5 mutations are also related to epileptic encephalopathy or intellectual disability [26]. In this scenario, cancer also alters the expression of Kv7 isoforms. In the present work, we show for the first time that the expression of Kv7 channels is remodeled in vascular cancer. Kv7.1 and Kv7.5 channels are expressed not only in vascular smooth muscle cells but also in the endothelial lining of human veins and arteries; thus, they may exert a role in endothelial-derived cancers. Vascular tumors, however, lack muscle layers, thus losing the contraction ability. Therefore, if Kv channels are found in these cancers, we expect them to perform a different role, probably related to proliferation or cell survival, instead of regulating vascular tone.

Since ion channel research in cancer started more than 40 years ago, many channels have been studied. However, Kv7 channels have received little attention. In this scenario, colon cancer was found to express elevated Kv7.1 levels, and thromboxane A2-induced proliferation increased Kv7.1 abundance [15]. Similarly, lung adenocarcinoma cells augment Kv7.1 expression and, by blocking channel activity, arrest in the G0/G1 phase of the cell cycle [17]. This result is consistent with the antiproliferative effect of linopirdine, a Kv7.5 inhibitor, on myoblast proliferation [18]. However, canine osteosarcoma cell proliferation decreases the expression of Kv7.5 [19]. Furthermore, tamoxifen, which efficiently inhibits Kv7.2/Kv7.3 activity, is used in breast cancer therapy [27]. All this evidence suggests that Kv7 channels are remodeled by cancer, mostly through increasing their expression. Our data are in agreement with this evidence, which indicates that indolent tumors express less Kv7.1 and Kv7.5 than their malignant counterparts. However, the heterogeneity found by these few studies suggests further work is warranted.

Kv7.1 and Kv7.5 associate in vascular muscle, and their relative expression differs among different types of vessels [14]. This heterogeneity can modulate the stoichiometry of the tetramer, probably leading to different functional properties of the complex. Although Kv7.1 concomitantly decreased with the indolence of the tumor, some cases presented a variable phenotype. A particular cavernous angioma specimen showed no changes in expression, affecting the overall conclusions. Furthermore, angiosarcoma biopsies, although presenting an elevated MI, exhibited a low TN Kv7.1 ratio. Kv7.5 expression correlated with malignancy. Kv7.5 participates in myoblast proliferation [18], and a higher proliferation in sarcomas and angiosarcomas is concomitant with a higher expression of Kv7.5. In contrast, downregulation of the channel could slow tumor progression, and pharmacological inhibition of Kv7.5 could halt proliferation.

Kv channels are considered potential biomarkers and targets for antitumor therapies. We demonstrated that Kv7 channels, which are essential for controlling vascular tone and are implicated in muscle cell proliferation, are expressed in endothelial cells and remodeled in tumor-associated vascular endothelial cancers. Our data suggest that there is good correlation between the level of channel expression and the malignancy of the tumor. Therefore, our work further supports ion channels as potential therapeutic targets in malignant vascular tumors.

## 4. Materials and Methods

### 4.1. Tissue Samples, Protein Extraction and Western Blot

All animal procedures were approved by the Ethics Committee of the University of Barcelona (86/609/EEC). Human protocols were approved by the ethics committee of the Catalan Institute of Oncology (Hospital Universitari de Bellvitge, PR074/11). Biopsies, deposited at the Banc de Teixits de l’Hospital Universitari de Bellvitge, required no further informed consent to use archived tumor samples.

Fresh rat tissues (heart, brain, and quadriceps) and human blood vessel (aorta, cava arteries and carotid and subclavian veins) biopsies were frozen in liquid nitrogen embedded in optimal cutting temperature compound (OCT). Prior to protein extraction, stock pieces were thawed in a saline solution. Tissue (250–500 mg) was cut and gently homogenized in 5 mL HES (20 mM HEPES ((4-(2-hydroxyethyl)-1-piperazineethanesulfonic acid), 1 mM EDTA (ethylene diamine tetraacetic acid), and 255 mM sucrose, pH 7.4) with protease inhibitors using a Polytron^®^ homogenizer. Nuclei and debris were removed by centrifugation at 15,000× *g* for 20 min at 4 °C. Supernatants were rehomogenized following the same procedure. Samples were incubated for 30 min at 4 °C and then were centrifuged at 200,000× *g* for 1.5 h at 4 °C. The supernatant was discarded, and pellet crude membranes were resuspended (0.5 mL/g tissue) in lysis buffer (5 mM EDTA, 5 mM EGTA (ethylene glycol tetraacetic acid), and 1% Triton X-100) with a 25 G needle. Protein concentration was quantified (Bradford, BioRad, Hercules, CA, USA) and frozen until later use.

Protein samples (50 mg) were boiled in Laemmli SDS loading buffer and separated on 10% SDS-PAGE gels. Samples were transferred to PVDF (polyvinylidene fluoride) membranes (Immobilon-P, Millipore, Burlington, MA, USA) and blocked in 5% dry milk with 0.05% Tween-20 in PBS (phosphate-buffered saline) before immunoreactions were performed. Membranes were immunoblotted with antibodies against Kv7.1 (1:500, Alomone, Jerusalem, Israel) or Kv7.5 (1:500, Alomone).

### 4.2. Fluorescence Immunohistochemistry in Human Samples

Human tumor and healthy samples were obtained from Banc de Teixits de l’Hospital Universitari de Bellvitge. Nontumor vessels used for this work were carotid arteria, aorta, subclavian vein and inferior cava vein. Tumor samples were from patients (age and sex not provided) with different types of vascular lesions. Paraffin-embedded tumor samples came from 24 cases containing 5 different tumor types (cavernous angioma (5), capillary hemangioma (5), Kaposi’s sarcoma (4), angiosarcoma (4), and glomus tumor (6)). In addition, OCT-embedded frozen samples were provided from 3 specimens and tumor types (cutaneous angiosarcoma (1), teratoma-derived angiosarcoma (1) and epithelioid haemangioendothelioma (1)). All samples were routinely stained with hematoxylin-eosin (HE) and examined by light microscopy.

Five-micrometer-thick sections of OCT-embedded frozen tissue samples were placed on poly-L-lysine-coated coverslips and kept at −80 °C until immunolabeling. Sections were fixed in 4% paraformaldehyde with 60 mM sucrose and permeabilized with 0.3% Triton X-100 in PBS-G (20 mM glycine and 0.05% Triton X-100 in PBS). After blocking for 1 h (5% nonfat powder milk, 10% goat serum, and 0.3% Triton X-100 in PBS-G), samples were incubated with anti-Kv7.1 (1:200, Alomone) or anti-Kv7.5 (1:200, Alomone) and anti-α-actin (1:500, Alomone) antibodies. Goat anti-mouse IgG coupled to Alexa Fluor 488 (1:500, Invitrogen, Waltham, MA, USA), goat anti-rabbit IgG coupled to cyanine 3 (1:200, Thermo Fisher Scientific, Waltham, MA, USA) and goat anti-rabbit IgG coupled to cyanine 5 (1:200, Thermo Fisher Scientific) were used as secondary antibodies. Nuclei were counterstained with DAPI (4′,6-diamidino-2-phenylindole, Sigma, St. Louis, MO, USA). Antigen peptides for Kv7.1 and Kv7.5 (Alomone) were used as negative controls.

Five-micrometer-thick sections of paraffin-embedded samples were stored at room temperature until immunolabeling. Sections were deparaffinized and rehydrated by batch-type dipping in solvents. Briefly, samples were subsequently soaked twice in xylene for 5 min, twice in 100% ethanol for 3 min, twice in 95% ethanol for 1 min, once in 70% ethanol for 3 min, once in 30% ethanol for 3 min and, finally, twice in PBS-G for 5 min. No antigen retrieval was required for either Kv7.1 or Kv7.5 staining. The rehydrated sections were immunostained following the abovementioned procedure used for OCT-embedded samples.

### 4.3. Confocal Scanning Laser Microscopy

For confocal sample analysis, two systems were alternatively used after sample preservation. Paraffin embedded samples were imaged under a Leica TCS SP5 II (2 photon) laser-scanning confocal spectral microscope (Leica Microsystems GmbH, Wetzlar, Germany) equipped with an argon multiline laser (458, 488 and 514 nm), a DPSS 561 nm laser and a helium-neon laser (633 nm). To screen the entire sample, Matrix HCS-A Software (Leica), a system that performs high content screening, was used. Briefly, a low magnification (10×) lens was used to take consecutive images of the whole sample size, which were then stitched together to generate a single image. This image was used to identify regions of interest (ROIs), including healthy and tumoral blood vessels. After they were identified, ROIs were individually set as the focal plane to avoid sample thickness variations. By using a high magnification lens (63× oil immersion objective lens (NA 1.32)), a new screening was performed for each ROI, and consecutive images were overlapped to obtain single representations of the area selected.

OCT-embedded samples were imaged under an LSM880 Zeiss confocal laser scanning microscope (Carl Zeiss Microscopy, Oberkochen, Germany). The process of image acquisition was similar to that described above, but in this case, no additional software was required, as the software has a function that allows the capture of consecutive images and automatic stitching to generate a single image.

In all cases, 10× images were taken at low resolution (256 × 256), 600 Hz speed, bidirectional mode scanning with a pinhole aperture of 2 Airy units at zoom 1. Scanning was performed sequentially for observation of each fluorescent protein by grouping UV and near red in one scan (DAPI and Cy3, 405 nm and 561 nm laser lines, respectively) and green and far red channels in another (Alexa 488 and Cy5, 458 nm and 633 nm line, respectively).

### 4.4. Histologic Analysis

The morphological features of tumor samples were evaluated in hematoxylin-eosin (HE)-stained slides. Mitotic figures were identified morphologically by the condensed “hairy” characteristics of the chromosomes to distinguish prophase, metaphase, anaphase and telophase stages. Mitosis was counted by direct evaluation of 10 high power fields (HPF) by using 400× magnification with a Leica DMIRB Inverted Leica Modulation Contrast Microscope (Leica). HPFs were randomly selected in the tumor area and surrounding regions covering up to a 1 mm^2^ distance. The total mitotic index was calculated as the ratio between the number of mitotic events and the total cell number per HPF and are represented as the mean percentage. A minimum of 1000 cells were counted from each tumor type.

## Supplementary Materials

The following are available online at https://www.mdpi.com/1422-0067/21/17/6019/s1.
