# Supplementary material for: Remodeling of Kv7.1 and Kv7.5 Expression in Vascular Tumors
Source: Int J Mol Sci. 2020 Aug 21;21(17):6019. doi: 10.3390/ijms21176019 (PMC7503939; doi:10.3390/ijms21176019)
Supplement: Supplementary file 1 [file ijms-21-06019-s001.pdf]

Supplementary Materials

Remodeling of Kv7.1 and Kv7.5 expression in vascular tumors

Clara Serrano-Novillo[1], Anna Oliveras[1], Joan Carles Ferreres[2], Enric Condom[3], Antonio Felipe[1]

[1]Molecular Physiology Laboratory, Dpt. de Bioquímica i Biomedicina Molecular, Institut de Biomedicina (IBUB), Universitat de Barcelona, Barcelona, Spain. [2]Consorci Corporació Sanitària Parc Taulí-Parc Taulí Hospital Universitari. Universitat Autónoma de Barcelona. [3] Institut d'Investigació Biomèdica de Bellvitge (IDIBELL). Hospital Universitari de Bellvitge.

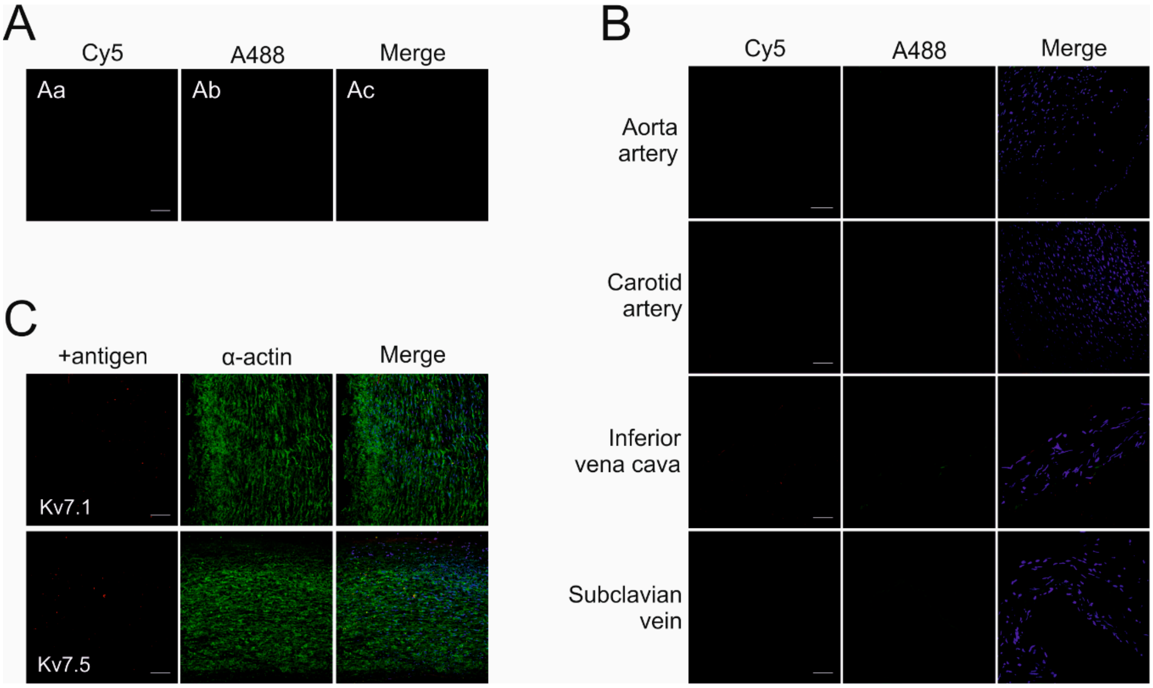

Supplementary Figure 1. *Negative controls for Kv7.1- and Kv7.5-specific staining in human veins and arteries.* Fresh human samples were obtained and processed as described in the Materials and Methods. (A) An autofluorescence control assessment with no antibodies was performed before each experiment. (Aa) Cy5 channel (633 nm); (Ab) Alexa488 α-actin (muscle) channel (458 nm). (Ac) merge Aa and Ab. Representative carotid artery experiment. Bar represents 50 µm. (B) Nonspecific fluorescence of secondary antibodies. Sections were stained with secondary antibodies in the absence of primary antibodies. Left panels, Cy5 (633 nm). Middle panels, Alexa488 (458 nm). Right panels show merge between Cy5 and Alexa488 with nuclear DAPI staining (405 nm, blue). Scale bars: 50 µm in inferior vena cava; 100 µm in carotid artery and subclavian vein; 150 µm in aorta artery. (C) Anti-Kv7.1- and anti-Kv7.5-specific staining. Controls were performed before each replicate of the experiment. Slices were incubated in the presence of the antigen peptide (+ antigen) following the manufacturer's instructions for Kv7.1 (top panels) and Kv7.5 (bottom panels). Left panels, channels in red. α-Actin identified muscle structures (middle panels, in green). Right panels show a merged image of red and green with nuclear DAPI staining. Scale bars: 100 µm.

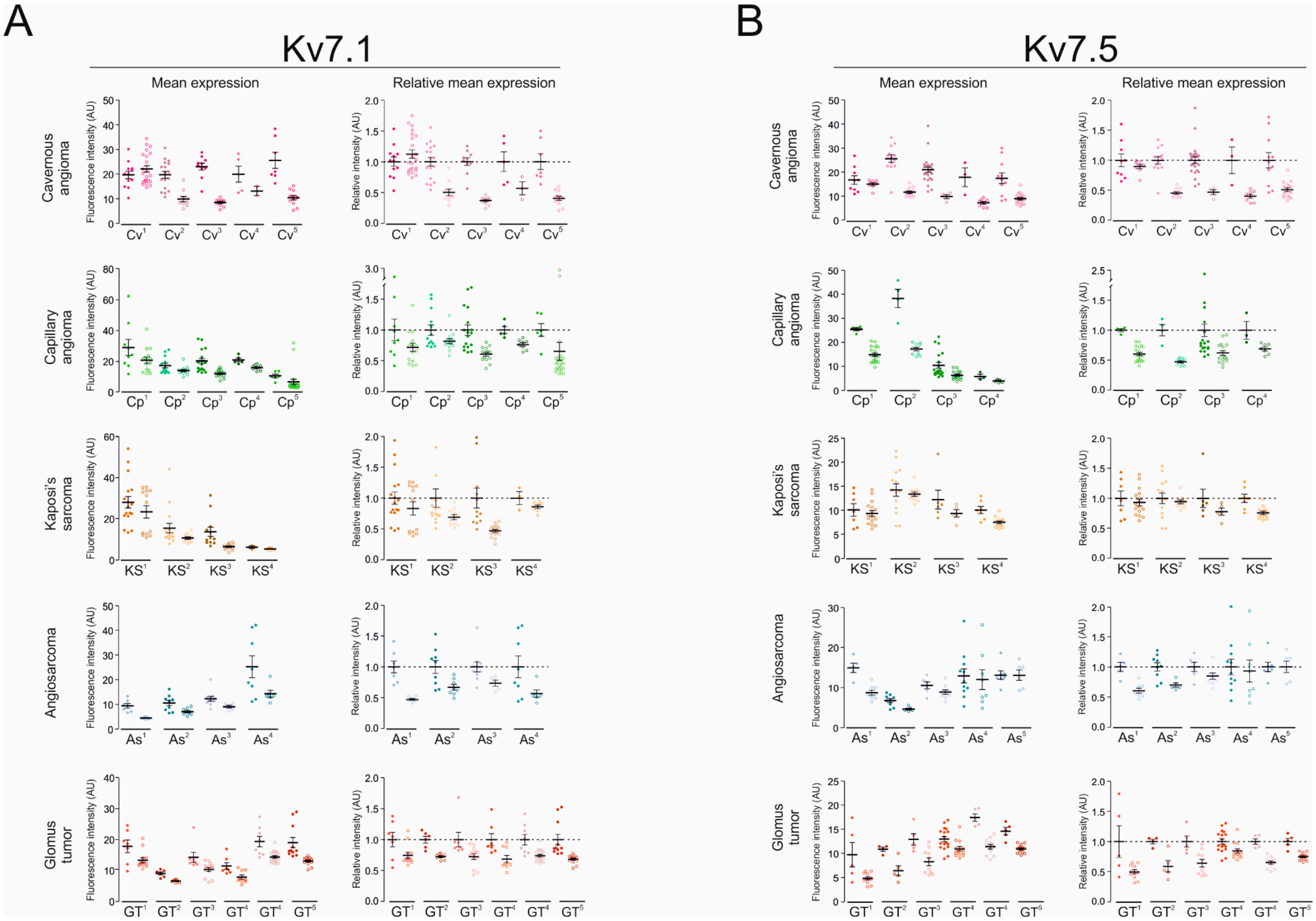
A
Kv7.1
Mean expression
Relative mean expression
Cavernous angioma
Capillary angioma
Kaposi's sarcoma
Angiosarcoma
Glomus tumor
Fluorescence intensity (AU)
Relative intensity (AU)
Cv¹ Cv² Cv³ Cv⁴ Cv⁵
Cp¹ Cp² Cp³ Cp⁴ Cp⁵
KS¹ KS² KS³ KS⁴
As¹ As² As³ As⁴
GT¹ GT² GT³ GT⁴ GT⁴ GT⁵
B
Kv7.5
Mean expression
Relative mean expression
Cavernous angioma
Capillary angioma
Kaposi's sarcoma
Angiosarcoma
Glomus tumor
Fluorescence intensity (AU)
Relative intensity (AU)
Cv¹ Cv² Cv³ Cv⁴ Cv⁵
Cp¹ Cp² Cp³ Cp⁴
KS¹ KS² KS³ KS⁴
As¹ As² As³ As⁴ As⁵
GT¹ GT² GT³ GT⁴ GT⁴ GT⁵

Supplementary Figure 2. *Kv7.1 and Kv7.5 expression in individual tumor biopsies.* Individual Kv7.1 (A) and Kv7.5 (B) expression levels from healthy blood vessels (closed symbols) and tumoral regions (open symbols) obtained from the immunostaining shown in Figure 2. Each dot corresponds to a single measurement for each individual. Individuals are indicated in superscript on the tumor abbreviation and are colored with different shades. Magenta, cavernous angioma (Cv); green, capillary angioma (Cp); orange, Kaposi's sarcoma (KS); blue, angiosarcoma (As); red, glomus tumor (GT). Horizontal lines show the mean ± SEM of each individual. The left panels show the mean fluorescence intensity. Right panels show the mean expression relative to the healthy control mean. A dotted line indicates reference control values (1).
